# Unveiling the Role of Sulfur Vacancies in Enhanced Photocatalytic Activity of Hybrids Photocatalysts

**DOI:** 10.3390/nano14121009

**Published:** 2024-06-11

**Authors:** Zhenxing Ren, Yang Li, Qiuyu Ren, Xiaojie Zhang, Xiaofan Fan, Xinjuan Liu, Jinchen Fan, Shuling Shen, Zhihong Tang, Yuhua Xue

**Affiliations:** 1 Institute of Applied Chemistry, Shanxi University, Taiyuan 030006, China; zxren@sxu.edu.cn (Z.R.);; 2 National & Local Joint Engineering Research Center for Mineral Salt Deep Utilization, Huaiyin Institute of Technology, Huaian 223003, China; 3 School of Materials and Chemistry, University of Shanghai for Science and Technology, Shanghai 200093, Chinajcfan@usst.edu.cn (J.F.);

**Keywords:** sulfur vacancies, photocatalysis, defect level, electron reservoir, adsorption and activation sites

## Abstract

Photocatalysis represents a sustainable strategy for addressing energy shortages and global warming. The main challenges in the photocatalytic process include limited light absorption, rapid recombination of photo-induced carriers, and poor surface catalytic activity for reactant molecules. Defect engineering in photocatalysts has been proven to be an efficient approach for improving solar-to-chemical energy conversion. Sulfur vacancies can adjust the electron structure, act as electron reservoirs, and provide abundant adsorption and activate sites, leading to enhanced photocatalytic activity. In this work, we aim to elucidate the role of sulfur vacancies in photocatalytic reactions and provide valuable insights for engineering high-efficiency photocatalysts with abundant sulfur vacancies in the future. First, we delve into the fundamental understanding of photocatalysis. Subsequently, various strategies for fabricating sulfur vacancies in photocatalysts are summarized, along with the corresponding characterization techniques. More importantly, the enhanced photocatalytic mechanism, focusing on three key factors, including electron structure, charge transfer, and the surface catalytic reaction, is discussed in detail. Finally, the future opportunities and challenges in sulfur vacancy engineering for photocatalysis are identified.

## 1. Introduction

Energy shortages and environmental pollution have emerged as pressing issues globally [1,2,3]. Photocatalysis has attracted considerable attention for its environmentally friendly characteristics, rooted in sustainability and renewability [4,5,6]. Various photocatalysts have been the subject of extensive research for applications in the nitrogen reduction reaction (NRR), carbon dioxide reduction reaction (CRR), hydrogen evolution reaction (HER), oxygen evolution reaction (OER), and air purification [7,8,9,10].

The photocatalytic reaction process involves three steps: (i) light absorption related to the band gap/defect level; (ii) charge transfer and separation related to the charge mobility, conductivity, defect level/conduction/valence position; and (iii) surface catalytic reaction related to the adsorption/desorption energy, active site number, and reaction activation energy [11,12]. The band gap of photocatalysts should meet the thermodynamic reaction potentials. The conduction/valence position of photocatalysts should align the potential of the adsorbate to facilitate the redox reaction process. Particularly, in the photocatalytic reduction process, most of the electrons and protons will combine to generate H_2_, rather than the CRR and NRR. The intense competition of HER on the photocatalyst’s surface results in unsatisfactory photocatalytic CRR and NRR activity. Promoting the OER reaction can provide additional electrons and protons for the CRR and NRR process. In these reactions, the absorption range of light and charge transfer are crucial factors for enhancing photocatalytic activity. Therefore, developing highly efficient photocatalysts is imperative [12,13]. 

Pure semiconductors suffer from lower light absorption, rapid recombination of photo-induced carriers, and poor surface catalytic activity for reactant molecules, resulting in reduced photocatalytic activity. Significant effort has been devoted to developing highly efficient photocatalysts, including heteroatom doping, co-catalyst loading, integrating different components, and more. Heteroatom doping can modify the energy band structure to enhance light absorption, while co-catalyst loading and the integration of different components can optimize the charge transfer at the interface and surface catalytic reactions, respectively [4,14]. 

It is widely recognized that the electronic structure of photocatalysts plays a crucial role in tuning their intrinsic photocatalytic activity. Surface defect engineering has been regarded as an effective strategy to adjust the electronic structure of photocatalysts to control light absorption and charge transfer, thereby enhancing the photocatalytic activity in processes such as CRR, NRR, OER, and HER [15,16,17]. Vacancies can manipulate the energy band structure to broaden the light absorption range and facilitate charge transfer, promoting the photocatalytic reaction [18]. By creating new defect energy levels and increasing the density of states around the Fermi level, vacancies can lead to a narrower band gap. Acting as electron traps, vacancies can prevent the vertical transmission of photo-induced electrons, accumulating electrons around [19] and enhancing the separation of charge carriers [20,21,22]. Additionally, vacancies can function as adsorption sites, reducing the adsorption energy of reaction molecules during photocatalytic process. Therefore, introducing sulfur vacancies can enhance the photocatalytic reaction of metal sulfides [23]. However, excess sulfur vacancies can act as traps that capture charges, altering the local electronic structure and inducing surface polarization. Thus, controlling surface sulfur vacancies presents an alternative and promising opportunity for photocatalytic reactions. Establishing and understanding the role of sulfur vacancies in photocatalysis is essential and pivotal.

It is widely known that defects exist in various dimensions, including point defects, such as doping and vacancies (both as bulk and surface defects), line defects (as bulk defects), planar defects (as bulk defects), and volume defects (as bulk and surface defects) [24,25]. Vacancies are considered as point defects in crystals. Both anions, such as oxygen, nitrogen, sulfur, iodine, and cations, such as Bi, Ti, C, and Zn vacancies, have been extensively studied in semiconductor photocatalysts [26,27,28,29]. The types and concentrations of vacancies in photocatalysts play a significant role in determining their photocatalytic activity. Various characterization methods, such as microscopes and spectroscopy techniques, can be utilized to verify the local distribution of vacancies and thereby clarify the structure–function relationship between vacancies and photocatalysis. Despite some reviews summarizing the photocatalytic activity of photocatalysts with sulfur vacancies, understanding the specific role of sulfur vacancies in photocatalysis still poses a challenge.

In this review, we aim to elucidate the pivotal role of sulfur vacancies in the photocatalytic reaction and offer valuable insights for the future development of high-efficiency photocatalysts enriched with sulfur vacancies. This review is structured into three primary sections: (i) establishing a fundamental understanding of photocatalysis, (ii) expounding on the engineering strategies and corresponding characterization methods for sulfur vacancies, and (iii) highlighting the significance of sulfur vacancies in enhancing photocatalytic activity, thereby clarifying the intricate structure–function relationship between vacancies and photocatalysis. Finally, we present the challenges and outlooks for sulfur vacancy engineering in photocatalysis.

## 2. Characterization and Engineering Strategies of Sulfur Vacancies

### 2.1. Characterization of Sulfur Vacancies in Photocatalysts

It is imperative to observe the atomic structures of photocatalysts with sulfur vacancies and comprehend the impact of these vacancies on photocatalytic activity. To achieve this, numerous characterization methods, encompassing microscopic and spectroscopic techniques, have been devised to validate the local distribution of vacancies [30,31,32]. Among these, microscope techniques, such as atomic-resolution spherical aberration-corrected transmission electron microscopy, scanning transmission electron microscopy (STEM), and scanning tunneling microscopy, are employed to verify the presence of vacancies [33]. Spectroscopy techniques, on the other hand, encompass electron spin resonance (ESR) spectroscopy, X-ray photoelectron spectroscopy (XPS), X-ray absorption fine structure (XAFS), in situ electron energy loss spectroscopy, transient absorption, Raman spectroscopy, thermogravimetric, time-resolved absorption, and fluorescence spectroscopy [34,35]. For instance, STEM, with the aid of probe-forming aberration correctors, can distinguish between surface and bulk vacancies, offering insights into local atomic structure and chemical composition at the atomic level. Additionally, annular dark-field (ADF) images obtained through STEM, pioneered by Liu et al., can verify the atomic structures and counts on an atom-by-atom basis, relying on the intensity of the ADF signal [36]. ESR spectroscopy is valuable in identifying defect species and quantifying defect density [37]. Meanwhile, XPS and XAFS analyses can verify the chemical environment and local atomic structures through peak shifting, variations in peak intensity, or the emergence of new peaks [38,39]. Therefore, these microscopic and spectroscopic characterization methods are crucial and indispensable in providing valuable insights into the structure–function relationship of vacancies, which are classified accordingly.

#### 2.1.1. High-Resolution Transmission Electron Microscopy 

**Figure 1:**
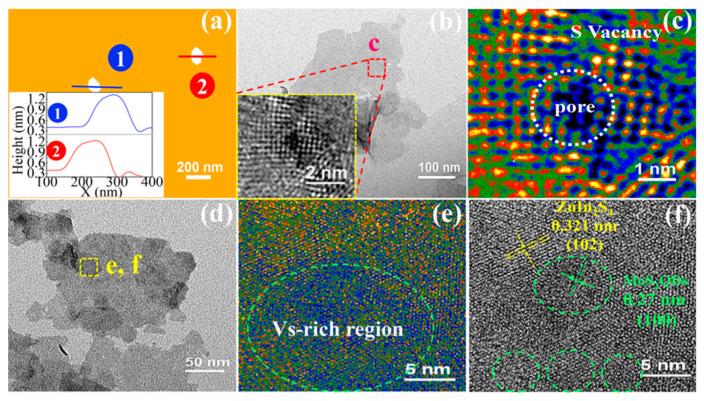
(**a**) AFM and (**b**,**c**) HRTEM images of Vs-M-ZnIn_2_S_4_ [19]; (**d**–**f**) HRTEM images of MoS_2_ quantum dots@Vs-M-ZnIn_2_S_4_. Green dashed circles represent the MoS_2_ QDs [19]. Reprinted with permission from Ref. [19]. Copyright {2018} American Chemical Society.

To verify the formation of sulfur vacancies, high-resolution transmission electron microscopy (HRTEM) is utilized, yet it lacks the capability to differentiate between surface and bulk vacancies. As depicted in Figure 1a,b, atomic force microscopy (AFM) and HRTEM images of the S vacancies in monolayered ZnIn_2_S_4_ (Vs-M-ZnIn_2_S_4_) reveal a nanostructure composed of single-layer sheets [19] (Table 1). The false-color HRTEM image of Vs-M-ZnIn_2_S_4_ in Figure 1c confirms that some atoms were absent (sulfur vacancies), and pores formed in Vs-M-ZnIn_2_S_4_ owing to the strong impact of the generated H_2_ [19]. Furthermore, a rich sulfur vacancies area can also observed in MoS_2_ quantum dots@Vs-M-ZnIn_2_S_4_ (Figure 1d–f) [19].

#### 2.1.2. Scanning Transmission Electron Microscopy

**Figure 2:**
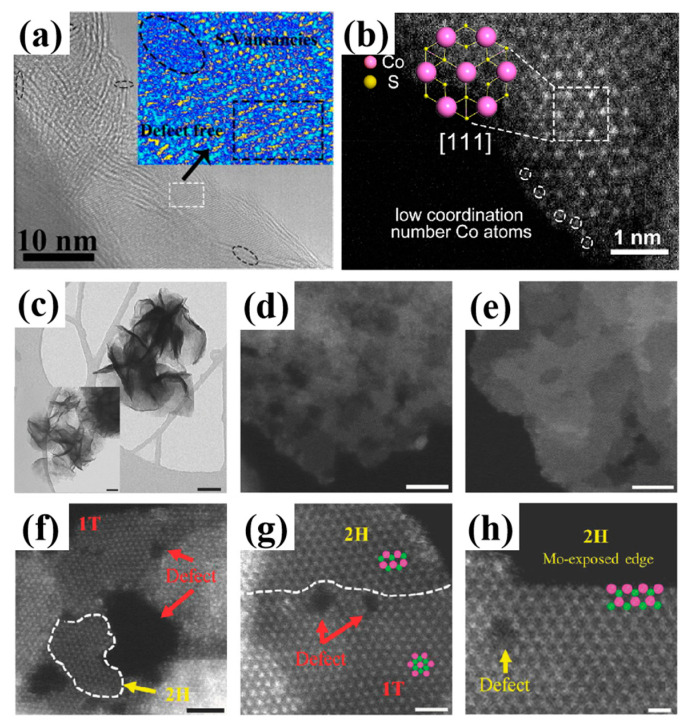
(**a**) Spherical aberration-corrected STEM image of MoS_2−x_@CdS [44]; (**b**) HAADF image of Co_3_S_4_ ultrathin porous nanosheets with rich sulfur vacancies [51]; Reprinted with permission from Ref. [51]. Copyright {2018} American Chemical Society; (**c**–**e**) bright-field HRTEM images of MoS_2_ [52]; (**f**–**h**) STEM images of MoS_2_ [52]. Reprinted with permission from Ref. [52]. Copyright {2018} Wiley. Dashed circles represent the sulfur vacancies. Scale bar = 50 nm (**c**), 5 nm (**d**,**e**), 2 nm (**f**) and 1 nm (**g**,**h**). Pink and green dots represent the Mo and S atoms, respectively.

Sulfur vacancies can be also characterized by STEM with probe-forming aberration correctors. Such techniques enable the differentiation between surface and bulk vacancies, providing detailed information on the local atomic structure and chemical composition at the atomic scale. In Figure 2a, the crystal fringes discontinued due to the abundant vacancies in the MoS_2−x_@CdS nanocomposite [44] (Table 1). In the inset image of Figure 2a, no defects can be detected in the rectangular area, while disordered atoms are observed in the circled area, indicating the presence of sulfur vacancies in MoS_2−x_@CdS.

An atomic-level high-angle annular dark-field (HAADF)-STEM image of Co_3_S_4_, depicting rich sulfur vacancies, is shown in Figure 2b [51]. The sulfur vacancies in Co_3_S_4_ ultrathin porous nanosheets stemmed from the reduction from Co^3+^ to Co^2+^. Notably, there is abundant low-coordination Mo atoms at the edges of the nanosheets and pores, suggesting increased disorder due to the presence of sulfur vacancies.

After thermal reduction, MoS_2_ exhibits a flower-like morphology characterized by sharp edges, larger flake sizes, and smaller holes, as depicted in Figure 2c–e. Figure 2f,g reveal the co-existence of 1T and 2H phases in MoS_2_, alongside nanoscale defects. Furthermore, the MoS_2_ layers exhibit small holes and well-defined Mo-terminated sharp edges in Figure 2h, which are similar to those of MoS_2_ synthesized by chemical vapor deposition, then exfoliating using lithiation and *n*-BuLi. Therefore, MoS_2_ retains nanoscale defects and sulfur vacancies following thermal reduction [52].

**Figure 3:**
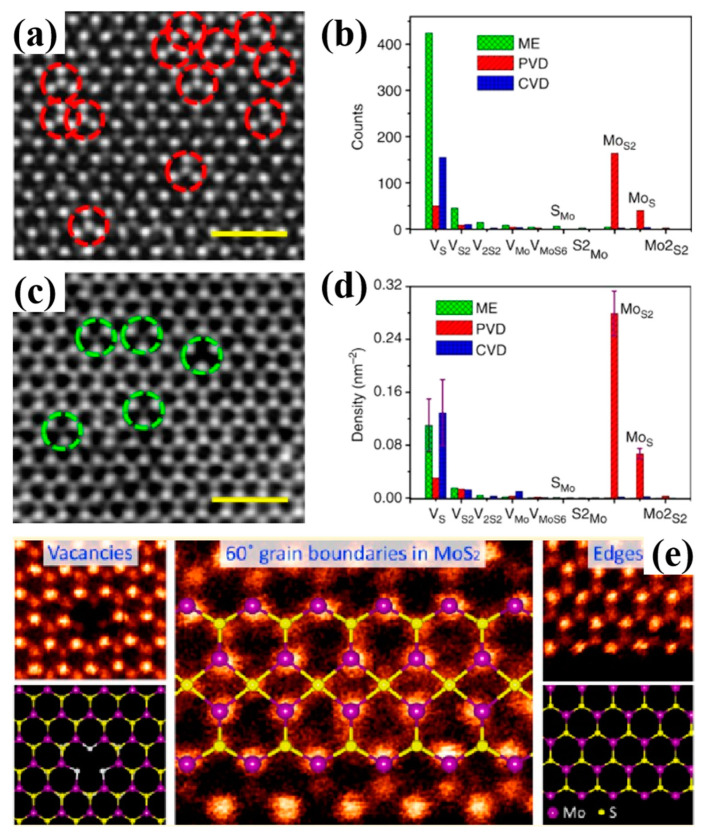
Atom-resolved STEM-ADF images of defects in MoS_2_ synthesized by (**a**) PVD and (**b**) ME [53]; (**c**,**d**) histograms of point defects in MoS_2_ [53]; Reprinted with permission from Ref. [53]. Copyright {2015} Nature/Scientific Reports; (**e**) STEM-ADF images of intrinsic point defects in MoS_2_ monolayer synthesized by CVD [54]. Scale bar = 1 nm (**a** and **c**). Red dashed circles represent Mo_S_ or Mo_S2_. Green dashed circles represent V_S_ or V_S2_. Reprinted with permission from Ref. [54]. Copyright {2013} American Chemical Society.

ADF images with STEM, introduced by Liu et al., are capable of verifying atomic structures and counts on an atom-by-atom basis, depending on the intensity of the ADF signal [36]. Defects in MoS_2_ synthesized by mechanical exfoliation (ME), chemical vapor deposition (CVD), and physical vapor deposition (PVD) are observed via atomically resolved ADF-STEM (Figure 3a,b) [53]. The ADF-STEM image of MoS_2_ monolayers synthesized via CVD is similar to that of ME MoS_2_ monolayers. Within the red dashed circles in Figure 3a, Mo atoms are obverted to replace either one S atom (Mo_s_) or two S atoms (Mo_S2_). Conversely, within the green dashed circles in Figure 3b, one or two sulfur atoms are absent, leading to the generation of sulfur vacancies in the ME and CVD samples, corroborating previously reported findings regarding CVD MoS_2_ monolayers, as shown in Figure 3e [54]. In Figure 3c,d, the predominant point defects of ME and CVD MoS_2_ monolayers are sulfur vacancies, with a concentration of (1.2 ± 0.4) × 10^13^ cm^−2^. In contrast, the predominant point defects of PVD MoS_2_ monolayers are Mo_S2_ and Mo_s_, with concentrations of (2.8 ± 0.3) × 10^13^ cm^−2^ and 7.0 × 10^13^ cm^−2^, respectively.

#### 2.1.3. Electron Spin Resonance

ESR spectroscopy can provide useful information for identifying defect species and verifying the defect density [37]. The ESR signal is attributed to the presence of unpaired electrons in materials. The increased intensity of the ESR signal correlates with the defect density, indicating a higher concentration of electrons being captured by sulfur vacancies.

**Figure 4:**
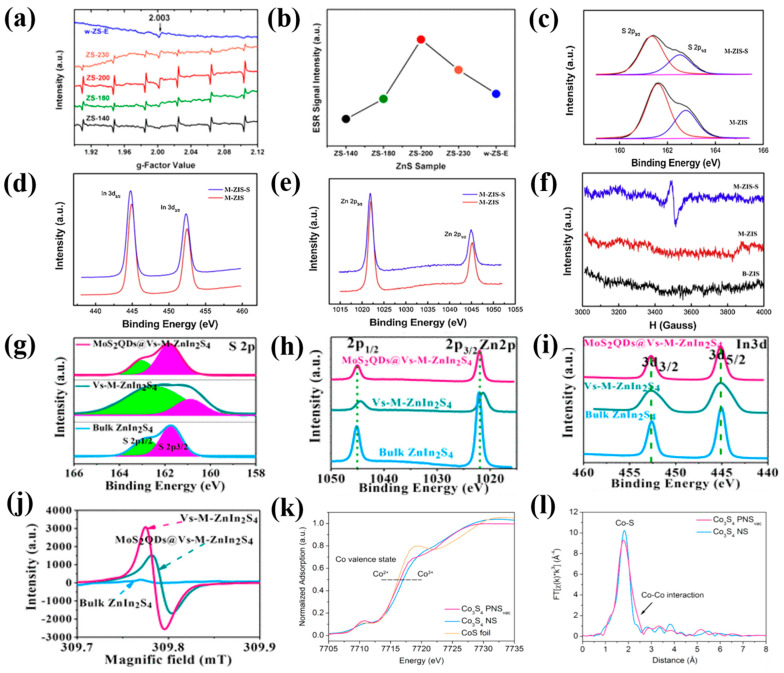
(**a**) ESR spectra and (**b**) intensity for ZnS [41]; Reprinted with permission from Ref. [41]. Copyright {2015} American Chemical Society; (**c**) S 2p, (**d**) In 3d, and (**e**) Zn 2p XPS spectra of M-ZIS-S [40]; Brown and blue lines represent S 2p1/2 and S 2p3/2, respectively. Black and pink lines represent S 2p XPS spectrum and base line, respectively; (**f**) ESR spectra of M-ZIS-S [40]; Reprinted with permission from Ref. [40]. Copyright {2019} Elsevier; (**g**) S 2p, (**h**) Zn 2p, and (**i**) In 3d XPS spectra in Vs-M-ZnIn_2_S_4_ [19]; Green and pink areas represent S 2p1/2 and S 2p3/2, respectively. (**j**) EPR spectra of Vs-M-ZnIn_2_S_4_ [19]; Reprinted with permission from Ref. [19]. Copyright {2018} American Chemical Society; (**k**) Co K-edge XANES and (**l**) Co EXAFS spectra of Co_3_S_4_ with sulfur vacancies [51]; Reprinted with permission from Ref. [51]. Copyright {2018} American Chemical Society.

In Figure 4a,b, the ESR measurement results indicate the amount of sulfur vacancies in ZnS depends on the reaction temperature [41] (Table 1). At the lower reaction temperature, ZnS undergoes gradual atomic rearrangement, with only a small quantity of the wurtzite phase forming initially. This results in the presence of sulfur vacancies in the initial wurtzite phase. When the temperature goes up to 180 °C, the sphalerite–wurtzite phase transformation becomes easier and faster, resulting in a large number of sulfur vacancies. Therefore, the amount of sulfur vacancies increases with the increased reaction temperature. When the reaction temperature increases to 200 °C, the sulfur vacancies concentration reaches the maximum. However, at even higher temperatures (i.e., 230 °C), all ZnS nanoparticles are completely transferred into the wurtzite phase, showing the crystal’s perfection, which decreases the amount of sulfur vacancies.

#### 2.1.4. X-ray Photoelectron Spectroscopy

XPS analysis can also be employed to verify the presence of sulfur vacancies on the surface of materials. These sulfur vacancies can alter the electron structure and chemical environment of elements, leading to XPS peak shifts, variations in XPS peak intensity, or the generation of new XPS peaks. In Figure 4c–e, compared with the ZnIn_2_S_4_ monolayer without sulfur vacancies (M-ZIS), the binding energies of S 2p, In 3d, and Zn 2p in the ZnIn_2_S_4_ monolayer with sulfur vacancies (M-ZIS-S) are lower, indicating the existence of sulfur vacancies [40] (Table 1). The intensity ratios of S 2p in M-ZIS and M-ZIS-S are smaller than those of In 3d and Zn 2p, providing further evidence for the existence of sulfur vacancies [40]. This is consistent with the ESR results in Figure 4f.

Similar results were also observed for Vs-M-ZnIn_2_S_4_ nanosheets (Figure 4g–i) [19]. The lower binding energy shift of Zn 2p in the Vs-M-ZnIn_2_S_4_ resulted in decreased electron state density around Zn atoms, suggesting that sulfur atoms are predominantly lost around Zn atoms in Vs-M-ZnIn_2_S_4_. However, when the MoS_2_ quantum dots were grown onto Vs-M-ZnIn_2_S_4_, the binding energy of Zn 2p increased in MoS_2_ quantum dots@Vs-M-ZnIn_2_S_4_, indicating that the sulfur content of the MoS_2_ quantum dots@Vs-M-ZnIn_2_S_4_ increased again. The ESR response results in Figure 4j also indicate that the sulfur vacancies concentration in MoS_2_ quantum dots@Vs-M-ZnIn_2_S_4_ was lower than that of Vs-M-ZnIn_2_S_4_. This was likely due to the vacancies of Vs-M-ZnIn_2_S_4_ being partially sealed by the sulfur originating from MoS_2_ quantum dots [19].

#### 2.1.5. X-ray Absorption Fine Structure

XAFS is utilized to probe the chemical environment and local atomic structures [38,39]. From the Co K-edge X-ray absorption near-edge structure (XANES) in Figure 4k, it can be found that the valence states of cobalt followed the order of CoS < Co_3_S_4_ with sulfur vacancies < Co_3_S_4_ without sulfur vacancies, indicating the presence of sulfur vacancies in Co_3_S_4_ [51]. For the extended X-ray absorption fine structure (EXAFS) spectra in Figure 4l, the peak intensity and width at ~1.84 Å followed the order of Co_3_S_4_ with sulfur vacancies < Co_3_S_4_ without sulfur vacancies. By fitting the EXAFS spectra, it can be determined that the coordination numbers and bond distance of Co-S in Co_3_S_4_ with sulfur vacancies decreased, indicating increased disorder degrees. The bond distance of Co-S for Co_3_S_4_ with and without sulfur vacancies contracted by 4% [51]. Similar results were observed for MoS_2_ [55], Ni_3_S_2_ [45] (Table 1), and FeS_2_/CoS_2_ [56].

### 2.2. Engineering Strategies for Sulfur Vacancies Generation

Sulfur vacancies can be generated by various methods, such as the lithiation-chemistry approach, thermal treatment, chemical reduction, and electrochemical treatment [57,58]. Understanding the formation of sulfur vacancies and their relative mechanism in the photocatalytic process is pivotal and essential.

#### 2.2.1. Lithiation-Chemistry Approach

**Figure 5:**
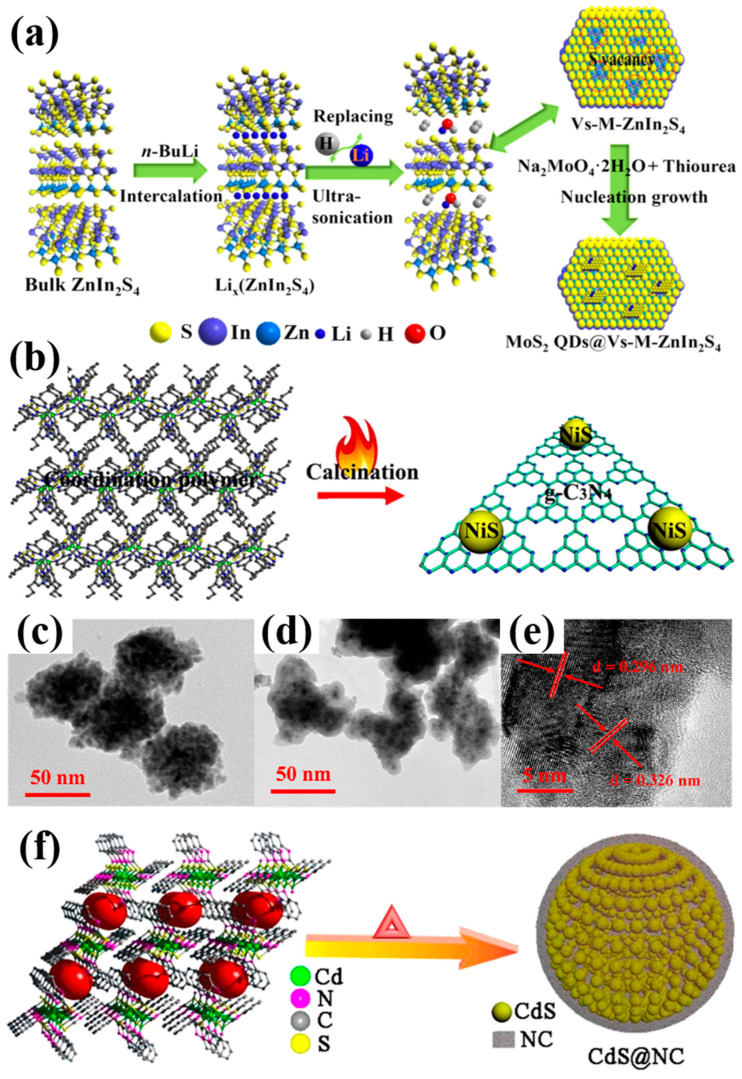
Formation mechanism of (**a**) MoS_2_ quantum dots@Vs-M-ZnIn_2_S_4_ [19] and (**b**) NiS@g-C_3_N_4_ [42]; Reprinted with permission from Ref. [19]. Copyright {2018} American Chemical Society; (**c**–**e**) HRTEM images of *g*-C_3_N_4_@NiS [42]; Reprinted with permission from Ref. [42]. Copyright {2018} American Chemical Society; (**f**) Synthesis process of CdS@NC composites with coordination polymer as a precursor [62]. Red dashed circles represent the sulfur vacancies. Reprinted with permission from Ref. [62]. Copyright {2018} American Chemical Society.

The lithiation-chemistry approach, as a promising strategy, has been developed to introduce sulfur vacancies [59,60]. For instance, sulfur vacancies on ultrathin ZnIn_2_S_4_ nanosheets are formed by exfoliating bulk ZnIn_2_S_4_ with the aid of lithium intercalation using *n*-butyllithium, which serve as a 2D platform for depositing MoS_2_ quantum dots due to the electrostatic attraction between the sulfur vacancies and MoO_4_^2−^, as shown in Figure 5a [19].

During the lithium intercalation process, the Li_x_ZnIn_2_S_4_ intermediate precursor with weakened layer interaction is obtained, which is crucial for the construction of sulfur vacancies. The Li_x_ZnIn_2_S_4_ intermediate precursor undergoes a hydrolysis reaction (Equation (1)) by the replacement of Li with an H atom in H_2_O, resulting in ultrathin ZnIn_2_S_4_ with rich sulfur vacancies. This is confirmed by HRTEM, XPS, and ESR.

Li_x_ZnIn_2_S_4_ + H_2_O → H_2_ + LiOH + V_s_-M-ZnIn_2_S_4_ (1)

#### 2.2.2. Thermal Treatment

The thermal treatment of metal-based coordination polymers at high temperatures has been developed to produce the sulfur vacancies [61]. Metal-based coordination polymers typically include metal ions (such as Ni^2+^ and Cd^2+^) and thiol nitrogen heterocyclic ligands, such as 2-mercapto-5-propylpyrimidine (MPPI), 2-mercaptobenzimidazole (MEBMI), 4,6-dimethyl-2-mercaptopyrimidine (DMMPY), and 3-mercapto-4-methyl-1,2,4-triazole (MMTZ) [46] (Table 1). During the calcination process, the growth of metal sulfides is restricted by the surrounding thiol nitrogen heterocyclic ligands, resulting in the formation of small metal sulfide nanoparticles. Additionally, some coordinated nitrogen atoms can replace S^2−^ in metal sulfides to generate sulfur vacancies.

For example, *g*-C_3_N_4_@NiS photocatalysts with sulfur vacancies were synthesized by calcinating the Ni^2+^-based coordination polymer [Ni(MPPI)_2_]*_n_*, as shown in Figure 5b [42] (Table 1). In Figure 5c–e, small NiS nanoparticles with a diameter of 6–8 nm dispersed in *g*-C_3_N_4_ are shown. During the preparation process, N atoms in Ni(MPPI)_2_] can replace S^2−^ ions in NiS and occupy their sites, leading to the production of sulfur vacancies in the *g*-C_3_N_4_@NiS. The content of sulfur vacancies depends on the calcination temperature. The ESR response results indicate that, when the calcination temperature increases from 500 °C to 600 °C, the sulfur vacancies concentration decreases, leading to a higher binding energy of S 2p_3/2_. A CdS@carbon matrix (NC) with rich sulfur vacancies was synthesized using [Cd(MEBMI]*_n_*·*n*(H_2_O) as the precursor by calcination, as shown in Figure 5f [62]. After calcination, N atoms can replace the sulfur atoms in CdS, leading to the production of sulfur vacancies.

#### 2.2.3. Chemical Reduction

**Figure 6:**
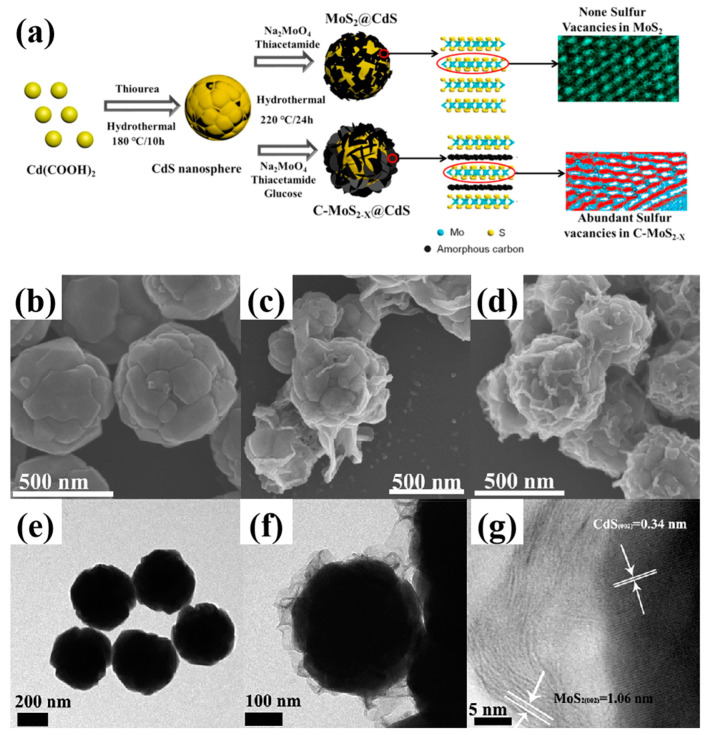
(**a**) Schematic of the preparation process; (**b**–**d**) SEM and (**e**–**g**) TEM images of MoS_2−x_@CdS and MoS_2_@CdS nanocomposites [44]. Reprinted with permission from Ref. [44]. Copyright {2020} Elsevier.

Chemical reduction methods, such as the introduction of reducing agents or using the reducing environment in the chemical reaction process, can induce the generation of vacancies. Reducing agents include hydrazine hydrate, NaBH_4_, KBH_4_, CaH_2_, N_2_H_4_, glucose, Al powder, Zn powder, and reducing solvents, such as ethylenediamine, ethanol, methanol, and ethylene glycol [45]. It is worth noting that chemical reduction methods typically only generate sulfur vacancies on the surface of photocatalysts. For example, glucose can act as a reducing agent in the prepared MoS_2−x_@CdS (Figure 6a) [44]. In Figure 6b–g, layers of amorphous carbon and MoS_2−x_ nanosheets are uniformly dispersed on the surface of CdS nanospheres. During the process, Mo^4+^ ions are partially reduced to Mo^3+^, resulting in the formation of sulfur vacancies on the MoS_2−x_ nanosheets’ surfaces.

The amount of sulfur vacancies can be adjusted by varying the amount of NaBH_4_ during the hydrothermal reaction process with concentrated NaOH solution. For ZnS, NaBH_4_ can reduce H_2_O to generate hydrogen, which in turn reduces the Zn^2+^ ions of the ZnS crystal lattice to form Zn^0^. The reduction also decreases the number of sulfur atoms in the ZnS crystal lattice, resulting in the formation of sulfur vacancies [63]. The concentration of NaBH_4_ determines the amount of sulfur vacancies. With an increased NaBH_4_ concentration, the amount of sulfur vacancies increases.

A reducing environment, such as H_2_, CO, NH_3_, and H_2_S, is beneficial for the formation of vacancies in the synthesis process [64]. For example, the hydrogenation method induces surface defects to obtain hydrogenated metal sulfide. Hydrogenated ZnIn_2_S_4_ is obtained by a pressure hydrogenation process in a home-built hydrogenation furnace connecting to the vacuum system, which is filled with hydrogen at 2.0 MPa and 300 °C [65]. Un-hydrogenated and hydrogenated ZnIn_2_S_4_ samples showed petaloid microspheres consisting of petal-like nanosheets. The hydrogenation process minimally alters the morphological and crystal structures of ZnIn_2_S_4_. Many surface defects of sulfur vacancies exist in hydrogenated ZnIn_2_S_4_ during the hydrogenation process.

Excess sulfur source can be adsorbed on the nanocrystal surface during the chemical reaction process, partially hindering the growth of crystals and leading to the formation of sulfur vacancies [66,67]. For example, increasing the concentration of thioacetamide sulfur source from 1.6 mmol to 3.2 mmol during the preparation process results in the formation of sulfur vacancies in ZnIn_2_S_4_ [40]. Further increasing the thioacetamide concentration from 4 mmol to 8 mmol leads to the formation of sulfur-vacancies-rich ZnIn_2_S_4_. Excess sulfur source induces the formation of defect structures [68]. 

Through adjusting the molar ratio of metal precursors for ternary metal sulfide, such as AgGaS_2_, silver and sulfur vacancies can be introduced to AgGaS_2_ nanocrystals. The EPR results indicate that the amount of sulfur vacancies increases with a decreased Ag/Ga molar ratio [69]. Furthermore, through adjusting the metal precursor, the amount of sulfur vacancies in metal sulfides, such as MoS_2_ and Cu*_x_*S, can be controlled [70]. For example, using (NH_4_)_2_MoS_4_ as a metal precursor results in MoS_2_ with fewer layers and more abundant unsaturated sulfur atoms at the external edge due to its quasi-amorphous structure during the process of crystal growth [43] (Table 1). However, sulfur vacancies in MoS_2_ using the Na_2_MoO_4_ precursor are not observed. 

## 3. The Role of Sulfur Vacancies in Photocatalysis

### 3.1. Defect Energy Levels

**Figure 7:**
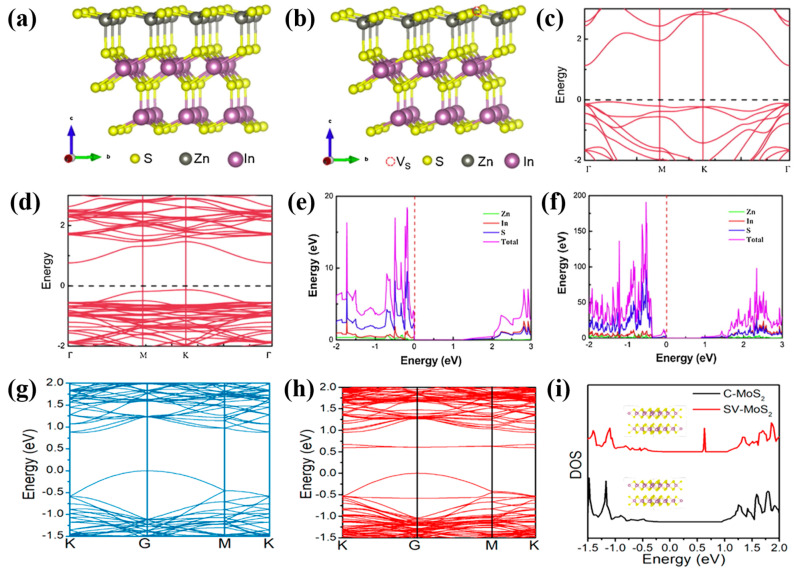
(**a**,**b**) Geometric structure, (**c**,**d**) band structure, and (**e**,**f**) DOS of M-ZIS and M-ZIS-S [40]; Reprinted with permission from Ref. [40]. Copyright {2019} Elsevier; Band structures of MoS_2_ without (**g**) and with (**h**) sulfur vacancies; (**i**) DOS for MoS_2_ with sulfur vacancies [48]; Reprinted with permission from Ref. [48]. Copyright © 2019 American Chemical Society.

The effect of sulfur vacancies on the electron structure of metal sulfide has been studied using density functional theory calculations. In Figure 7a,b, the introduction of sulfur vacancies to M-ZIS greatly alters its electron structure [40]. In Figure 7c,d, the band gap (2.31 eV) of M-ZIS-S is smaller than that of the M-ZIS (2.39 eV). Sulfur vacancies can lead to the formation of a defect energy band (−1.38 eV vs. NHE) located above the valence band maximum and below the conduction band minimum [42]. In Figure 7e,f, sulfur orbitals predominate in the conduction band minimum and valence band maximum in M-ZIS and M-ZIS-S. The density of states (DOS) in M-ZIS-S is higher than that in M-ZIS, indicating an increase in carriers, particularly at the conduction band minimum. The conduction band minimum of both M-ZIS and M-ZIS-S is primarily dominated by the 5s5p and S 3p orbitals. In M-ZIS, the valence band maximum is primarily dominated by the S 3p orbital, while in M-ZIS-S, both the S 3p and Zn 3p orbitals contribute to the valence band maximum. The valence band potentials of bilayer ZnIn_2_S_4_, M-ZIS, and M-ZIS-S are 1.52, 1.61, and 1.86 eV, respectively. Regarding the conduction band, the potentials of bilayer ZnIn_2_S_4_, M-ZIS, and M-ZIS-S are −0.82 eV, −0.78 eV, and −0.45 eV, respectively. A similar result has also been observed for sulfur-vacancies-rich MoS_2_ [48] (Table 1). Therefore, the introduction of sulfur vacancies can greatly influence the electronic properties, leading to shifts in the band gap, valence band, and conduction band of metal sulfide. Therefore, sulfur-vacancies-rich metal sulfides hold potential as visible light-responsive photocatalysts. 

Furthermore, the sulfur vacancies in MoS_2_ can also modify the electron structure, as shown in Figure 7g–i [48]. Two unoccupied states as a new defect level are introduced at approximately 0.63 eV between the valence band and conduction band, resulting in a narrow band gap for MoS_2_. MoS_2_ with sulfur vacancies shows enhanced absorption in the infrared region. 

### 3.2. Electrons Reservoir

The photoluminescence intensity serves as a confirmation of charge recombination. A lower photoluminescence intensity indicates reduced recombination of charge in the photocatalysts. The average emission lifetime (*τ*) is calculated by fitting time-resolved photoluminescence decay spectra using Equation (2):

(2) $$\tau =\frac{A_{1}\tau_{1}^{2}+A_{2}\tau_{2}^{2}+A_{3}\tau_{3}^{2}}{A_{1}\tau_{1}+A_{2}\tau_{2}+A_{3}\tau_{3}}$$

The photocurrent rapidly decreases to zero when the light is switched off, indicating charge recombination. Conversely, the increased photocurrent and longer lifetime imply improved charge separation and transfer efficiency. A higher photocurrent suggests more efficient charge transfer and longer lifetime.

The semicircle observed in the electrochemical impedance spectroscopy spectra is attributed to the contribution from charge transfer resistance (R_ct_) and the constant phase element at the photocatalysts/electrolyte interface. The inclined line, arising from the Warburg impedance, corresponds to the ion diffusion in the electrolyte. The value of R_ct_ confirms the charge transfer efficiency. A lower R_ct_ value indicates more effective charge transfer in the photocatalytic process.

For M-ZIS-S with sulfur vacancies [40], it shows a weaker photoluminescence peak, longer average fluorescent lifetime (*τ*_A_ = 5.04 ns), and lower electron transfer resistance *R*_ct_ compared with the M-ZIS. The results indicate that the introduction of sulfur vacancies facilitates charge carrier separation. 

Compared with ZnIn_2_S_4_, hydrogenated [65] and monolayer ZnIn_2_S_4_ [19] with sulfur vacancies show stronger photocurrent intensity, indicating more efficient charge carrier separation and transfer processes. Similar results, such as the lower photoluminescence emission intensity, approximately double photoluminescence lifetime (*τ*_A_ = 67.38 ns), and small *R*_ct_ value, are observed for hydrogenated ZnIn_2_S_4_ [65]. Similar results are also found for SnS_2_ with sulfur vacancies [48]. 

Sulfur vacancies induce bonding and antibonding states in the band gap, which serve as trapping sites for charge carriers [61]. An appropriate amount of sulfur vacancies in photocatalysts can effectively trap electrons, thereby reducing electron accumulation, which is advantageous for the photocatalytic reaction process. However, excessive and uncontrollable sulfur vacancies may serve as recombination centers, promoting the recombination of photo-induced charge carriers. This phenomenon can lead to unfavorable effects on the photocatalytic process. The balance between these factors determines the overall effect of sulfur vacancies on the photocatalytic reaction process. 

### 3.3. Adsorption and Activate Sites

Sulfur vacancies on the photocatalysts’ surfaces can serve as adsorption and active sites, such as SO_3_^2−^/S^2−^ ions, Cr(VI), formaldehyde, and N_2_, promoting the photocatalytic reaction. Taking photocatalytic hydrogen production as an example, the enhanced photocatalytic mechanism with sulfur vacancies is indicated as follows.

**Figure 8:**
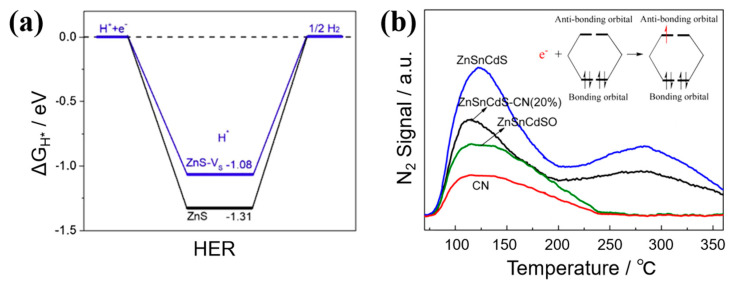
(**a**) Free energy diagram of perfect ZnS and ZnS with sulfur vacancies [66]; Reprinted with permission from Ref. [66]. Copyright {2020} Elsevier; (**b**) N_2_-TPD of g-C_3_N_4_(CN), ZnSnCdSO, ZnSnCdS, and ZnSnCdS-g-C_3_N_4_(CN)(20%) [50]; Reprinted with permission from Ref. [50]. Copyright {2016} American Chemical Society.

Sulfur vacancies play a beneficial role in hydrogen adsorption by altering the intermediate free energy (ΔG_H*_). In Figure 8a [66], the ΔG_H*_ (−1.08 eV) of ZnS with rich sulfur vacancies is smaller than that of perfect ZnS (−1.31 eV). Moreover, the hydrogen adsorption ΔG_H*_ depends on the amount of sulfur vacancies [71]. The optimal ΔG_H*_ value with activation energy is 0 eV, indicating that hydrogen is bound neither too strongly nor too weakly. In contrast, the ΔG_H*_ of pristine 2H-MoS_2_ is approximately 2 eV uphill. When sulfur vacancies are introduced into 2H-MoS_2_, ΔG_H*_ decreases with increasing sulfur vacancies concentration. Within the range of 9–19% for the sulfur vacancies concentration, the ΔG_H*_ is approximately ±0.08 eV, a value comparable to or even better than that of MoS_2_ with edge sites (0.06 eV [72]). With higher concentrations of sulfur vacancies, γ increases, indicating a less stable surface [71]. When ΔG_H*_ is 0 eV under the optimal condition, the sulfur vacancies concentration is about 11%. Therefore, the optimal sulfur vacancies concentration is estimated to be around 11%, resulting in maximal active site density and optimal catalytic activity.

Sacrificial reagents, such as Na_2_SO_3_/Na_2_S, lactic acid, organic amines, and alcohols, are added to the photocatalytic hydrogen production reaction process [61]. In the Na_2_SO_3_/Na_2_S system, sulfur vacancies on the photocatalysts surfaces can act as adsorption sites for S^2−^. The adsorption of SO_3_^2−^ ions on the photocatalysts’ surfaces is proven by the XRD patterns of CdZnS after the photocatalytic hydrogen reaction [73]. SO_3_^2−^ and S^2−^ ions can trap photo-induced holes, which is favorable for the charge transfer and photocatalytic reaction. The low oxidation potentials and high permittivity of SO_3_^2−^ and S^2−^ ions contribute to rapid hole consumption, thereby enhancing the hydrogen production rate and mitigating hole-induced photocorrosion. However, the sulfur ions in Na_2_SO_3_/Na_2_S repair the surface sulfur vacancy sites, leading to a decreased sulfur vacancies concentration on the photocatalysts’ surfaces, which is unfavorable for the photocatalytic hydrogen production reaction [65]. In lactic acid, organic amines and alcohols form a system with positive oxidation potentials and low permittivity and the holes are slowly consumed, resulting in a lower photocatalytic hydrogen production rate. 

Sulfur vacancies can also serve as active sites for adsorbing and activating N_2_ molecules [47] (Table 1). The nitrogen temperature-programmed desorption (N_2_-TPD) presented in Figure 8b shows strong chemisorption species of N_2_ at 270 °C for ZnSnCdS and g-C_3_N_4_(CN)-ZnSnCdS, but no peak is found for g-C_3_N_4_ and ZnSnCdSO [50] (Table 1). In addition, ZnSnCdS shows stronger physical adsorption of N_2_ at 120 °C [50]. The defects can serve as electron capture sites to enhance the adsorption on the surface of ZnSnCdS. This improvement facilitates interfacial charge transfer from g-C_3_N_4_ to N_2_ molecules, enhancing nitrogen photofixation activity. Specifically, the highest NH_4_^+^ generation rate for g-C_3_N_4_-ZnSnCdS with a ZnSnCdS mass percentage of 80% is 33.2-fold and 1.6-fold greater than those of pure g-C_3_N_4_ and ZnSnCdS, respectively. N_2_ molecules can undergo activation to ^*^N_2_ by donating electrons from bonding orbitals and accepting electrons into the three π^*^ antibonding orbitals. This process facilitates nitrogen photo-fixation and is driven by the correlation between adsorption and activation [74]. When N_2_ molecules adsorb on the sulfur vacancies in CdS, the N≡N bond length is obviously elongated from 1.164 Å to 1.213 Å, further demonstrating that the N_2_ molecules are activated by sulfur vacancies [49,75] (Table 1).

In summary, the role of sulfur vacancies in enhancing photocatalytic activity can be outlined as follows: (i) the introduction of sulfur vacancies to metal sulfide efficiently adjusts the electron structure, leading to the formation of a defect energy band located above the valence band maximum and below the conduction band minimum, which reduces the band gap of metal sulfide, resulting in excellent light absorption [76,77]; (ii) an appropriate amount of sulfur vacancies can act as the electrons reservoir to facilitate the charge transfer [76]; (iii) sulfur vacancies can increase the number of adsorption and active sites, such as SO_3_^2−^/S^2−^ ions, Cr(VI), formaldehyde, N_2_, and CO_2_, resulting in more photo-induced electrons being involved in the reduction reaction.

## 4. Conclusions and Perspectives

Sulfur vacancy engineering has demonstrated promising applications in enhancing the photocatalytic activity of metal sulfides, such as HER, CRR, and pollutant degradation. This enhancement is achieved by narrowing the band gap, facilitating charge transfer, and providing additional adsorption and activation sites. Despite significant improvements in photocatalytic activity, several major drawbacks still hinder practical applications. 

Firstly, effective controlling the types and structures of sulfur vacancies is crucial for enhancing the photocatalytic activity of metal sulfides. More preparation methods should be developed to regulate the types and structures of sulfur vacancies according to requirements.

Secondly, it is essential to investigate the influence of sulfur vacancies on other photocatalytic processes, such as CRR, Cr(VI) reduction, NRR, and OER. Detailed insights into the essential connections between sulfur vacancies and photocatalytic activity need to be organized to fully understand the role of sulfur vacancies in the photocatalytic process based on the combination of quantity theoretical calculations and experimental data. More characterization techniques, such as in situ XPS, in situ aberration-corrected electron microscopy, and in situ Raman spectroscopy, should be utilized to clarify the change in local electronic structures during the photocatalytic reaction process. This deepens our understanding of the effect of vacancies on the photocatalytic activity, providing scientific guidance for optimizing the photocatalytic activity through vacancies engineering.

Moreover, the poor stability of metal sulfides with sulfur vacancies in different sacrificial reagents remains poorly understood and requires further exploration to effectively address this limitation.

## Figures and Tables

**Table 1:** Summary of sulfur vacancy-rich photocatalysts for photocatalytic reaction.

Catalysts	Synthesis Methods	Characte- Rization Methods	Role of Defects	Tunable Properties	Refs.
ZnIn_2_S_4_	Exfoliation using n-butyllithium/ Hydrothermal processes	False-color HRTEM XPS EPR HAADF- STEM	Blue-shifted absorption edge Enhanced light absorption Efficient charge separation	Photocatalytic hydrogen evolution	[19,40]
ZnS	Hydrothermal processes	XPS ESR	New energy levels Efficient charge separation Active sites	Photocatalytic hydrogen evolution	[41]
NiS@g-C_3_N_4_	Calcination of coordination polymer	XPS ESR	Hydrophilicity with enhanced water adsorption Efficient charge separation	Photocatalytic hydrogen evolution	[42]
1T@2H MoS_2_	Hydrothermal processes	XPS EPR	H_2_O activation sites Efficient charge separation	Photocatalytic hydrogen evolution	[43]
MoS_2−X_@CdS	Hydrothermal processes	False-color STEM XPS EPR	Electronic reservoir Active sites	Photocatalytic hydrogen evolution	[44]
Zn_0.5_Cd_0.5_S_1−x_	Co-precipitation-hydrothermal strategy	XPS	Mid-gap impurity level Electron trapping site	Photocatalytic hydrogen evolution	[45]
CdS@3D-NPC	Calcination of coordination polymer	XPS EPR	Electron carriers VOC traps	Photocatalytic VOC removal	[46]
CdS/NCP	Thermal treatment	STEM XPS	CO_2_ adsorption sites Active sites Efficient charge separation	Photoelectrochemical reduction CO_2_	[47]
SnS_2_	Hydrothermal processes	ICP-AES XPS EPR HAADF-STEM	Efficient charge separation Cr(VI) adsorption sites Enhanced light harvesting	Photocatalytic reduction Cr(VI)	[48]
1T-MoS_2_@CdS	Hydrothermal processes	XPS ESR	Enhanced light absorption Improved electron separation Active edge sites	Photocatalytic Nitrogen Fixation	[49]
g-C_3_N_4_/ZnSnCdS	Hydrothermal processes	XPS	Active sites Improved electron separation	Photocatalytic Nitrogen Fixation	[50]

## Data Availability

Data are contained within the article.
